# Simulation of transcription factor clustering in nuclei from molecular kinetics

**DOI:** 10.64898/2026.06.04.730171

**Published:** 2026-06-06

**Authors:** Manya Kapoor, Mustafa Mir

**Affiliations:** 1 Department of Bioengineering, University of Pennsylvania; Philadelphia, PA 19104, USA.; 2 Center for Computational and Genomic Medicine, Children’s Hospital of Philadelphia; Philadelphia, PA 19104, USA.; 3 Department of Cell and Developmental Biology, Perelman School of Medicine, University of Pennsylvania; Philadelphia, PA 19104, USA.; 4 Howard Hughes Medical Institute, Children’s Hospital of Philadelphia; Philadelphia, PA 19104, USA.; 5 Epigenetics Institute, University of Pennsylvania Perelman School of Medicine; Philadelphia, PA 19104, USA.

## Abstract

Transcription factors form clusters often described as condensates that exhibit emergent biophysical properties. Here we present a software package to simulate transcription factor spatial distributions from molecular diffusion and binding kinetics alone. The software simulates microscopy data and FRAP experiments and recapitulates the clustering behavior of experimentally characterized transcription factors. Our results demonstrate that condensate-like structures can emerge from molecular kinetics principles without invoking higher-order processes like phase separation.

Transcription factors (TFs) are central to the regulation of gene activity. Two key observations have challenged canonical models of transcription factor function. First, *in vivo* molecular kinetics measurements have shown that TFs generally bind chromatin for just tens of seconds, inconsistent with the sustained burst of transcriptional activity that can last for orders of magnitude longer^1^. Second, the spatial distributions of TFs within the nucleus are generally clustered^2^. These clusters, often referred to as condensates or hubs, are proposed to be higher-order regulatory structures that promote sustained transcription factor occupancy by concentrating factors around targets^3,4^. Condensates are defined as non-stoichiometric assemblies proposed to form through phase separation mechanisms driven by multivalent protein interactions^5,6^. They are often characterized by emergent biophysical characteristics such as spherical shapes, fusion events, and rapid recovery after photobleaching^3,4,7,8^. However, recent measurements have suggested that the appearance of condensate-like bodies may be explained more simply by molecular diffusion and binding kinetics and limitations of microscopy^9-12^. Inspired by these observations, we developed a computational model to simulate the spatial distribution of TFs as they appear under microscope conditions based solely on molecular kinetics and explicitly excluding multi-valent protein-protein interactions. The output of this model can then be analyzed to gain intuition on whether emergent condensate properties can be predicted from diffusion and binding kinetics.

We have packaged this model in a user-friendly interactive tool dubbed SPARK (Simulation of Protein Accumulation from Reaction Kinetics). The software allows users to specify a range of experimentally-measurable molecular kinetic parameters which are used to generate molecular trajectories. Each molecule undergoes Brownian diffusion until it encounters a binding site, to which it can bind with a user-defined probability k_on_. Users can specify the number, clustering, and strength of binding sites, as well as introduce synthetic binding site arrays. The binding sites diffuse via fractional Brownian motion to recapitulate constrained polymer motion, and upon successful binding, the molecule diffuses along with the binding site. The duration of each binding event is drawn from a user-defined residence time distribution after which the molecule unbinds and resumes free diffusion (Fig. 1A).

The tool generates two visual outputs: “ground truth” images of molecular positions and binding sites, and simulated microscope images. The latter is produced by convolving the molecular position data with the microscope’s point spread function (PSF) and mapping the results to discrete pixel intensities, mimicking a digital camera sensor. This approach can be used to generate simulated time-lapse volumetric imaging data, taking into account variables such as z-slice spacing, time between frames, and exposure time. The ground truth and simulated microscope images can be viewed on a browser and downloaded as multi-page TIFFs for further analysis (Fig. 1B, C, Fig. S1). SPARK also includes a “batch processing” mode which allows users to input a range of parameters in a CSV and download results in bulk (Fig. S1). Additionally, the users can run simulations in parallel on a high performance computing cluster. Finally, as a commonly used experiment to test whether a cluster is a biomolecular condensate is Fluorescence Recovery After Photobleaching (FRAP), SPARK includes a module to perform computational FRAP experiments on regions of interest within simulated images.

We systematically modulated individual molecular kinetic parameters to gain intuition on how they can alter the appearance of clusters. We find that modulating the number and strength of binding sites at a single locus affects the enrichment of the TF over nuclear background at the locus, leading to the appearance of a bright, stable cluster (Fig. 1D). More globally, increasing TF concentration and overall number of binding sites in the nucleus increases the visibility and persistence of clusters (Fig. 1E). We find that similar clustering can be achieved by modulating either the on-rate (the probability that a molecule in the vicinity of a binding site will bind) or the residence time of the molecule (Fig. 1F). Importantly, altering microscope parameters (exposure time, resolution, etc.) can have a profound impact on how clusters appear with identical underlying molecular kinetics. For example lower spatial or temporal resolution can lead to the appearance of larger and more spherical clusters (Fig. S2).

To test the utility of SPARK in exploring real-world examples, we generated synthetic data for several well characterized transcription factors which are known to form clusters. We first examined Dorsal, a morphogen that forms a concentration gradient along the dorso-ventral axis of the *Drosophila* embryo. Using published data^10,13,14^, we simulated nuclei on the ventral, medial, and lateral sides of the embryo demonstrating that clustering can be recapitulated across all TF concentrations (Fig. 2A). Similarly, we simulated Bicoid, an anterior-posterior morphogen TF in the *Drosophila* embryo which is also known to cluster at both high and low nuclear concentrations^15-18^. Consistent with experimental observations, we found that more clusters are formed at higher nuclear concentrations in the anterior and the number of molecules within simulated clusters shows a linear relationship with concentration^19^ (Fig. 2B). However, the mean number of molecules per simulated cluster are lower than measured values. This discrepancy is likely due to the ability to segment smaller, dimmer clusters due to lower noise in simulated data or due to inaccurate nuclear concentration measurements in previous reports. To demonstrate SPARK’s ability to simulate super-resolution microscopy data we next examined the clustering of Sox2, a key pluripotency regulator known to have clustered binding sites^20^. Consistent with published data we find that Sox2 clusters only become visible upon clustering of its binding sites^21^ (Fig. 2C, Movie S1). Finally, to demonstrate that simulated data can recapitulate two key “emergent” condensate properties we show that clusters arising from DNA binding can (1) exhibit fusion and (2) show fluorescence recovery after photobleaching at similar timescales as those reported for condensates^3,8^ (Fig. 2D,E, Movie S2).

In conclusion, SPARK demonstrates that DNA binding and diffusion kinetics on their own are able to recapitulate the appearance and biophysical properties of condensate-like bodies formed by transcription factors. We conclude that clusters that might appear to be liquid-like condensates in fluorescence microscopy data can arise from molecular binding kinetics. It is important to note that SPARK does not account for protein-protein interactions, nor does it directly rule out higher-order processes of cluster formation. Instead, it asks whether observed clusters can be explained by a simple model of binding and diffusion kinetics without needing to invoke higher-order mechanisms for cluster formation. SPARK will allow users to similarly assess whether the clusters they observe can be explained by DNA binding and diffusion kinetics, rather than higher-order phenomena like condensate formation.

## Methods

### Simulator overview

SPARK is a browser-based tool implemented in Streamlit/Python, used to generate ground truth and "microscope-like” images from molecular data. The simulator consists of an interactive mode, which allows users to simulate and visualize results from a single set of parameters in the browser, and a batch mode where users can upload a CSV specifying a range of simulation parameters, the results of which can be downloaded. A single physics engine drives both modes. Additionally, a command-line implementation of the same engine enables running simulations on high-performance computing cores. Note that all simulated data are intended as helpful, intuition-building visualizations and not ground truth data.

#### Geometry and molecule count

The nucleus and transcription factor (TF) molecules are modelled as spheres. The number of TF molecules is set from the user-supplied bulk concentration and the nuclear volume:

$$N=\lfloor c\cdot 10^{-9}\cdot V_{nuc}\cdot 10^{-15}\cdot N_{A}\rfloor$$

with c in nM, $V_{nuc}$ in μm^3^ and *N_A_* = 6.022 × 10^23^. All distances internal to the engine are tracked in micrometres; nanometres are used for image-space parameters (PSF, voxel size, slice spacing).

#### Time stepping and exposure averaging

Three timescales coexist:

- **Physics step** *Δt*: the timestep at which positions, states, and binding events are updated. By default this is set at the exposure time for single molecule tracking experiments.
- **Exposure time** *T_exp_*: a contiguous block of *T_exp_/Δt* physics steps representing camera integration. At every step within the window, a snapshot of all molecule positions, states, site positions, and site occupancies is recorded.
- **Display interval** *T_disp_*: the period at which images are generated. Each display time has an exposure time window around it. The remaining physics steps are "dark" steps that advance the system without generating an image.

For each output frame, molecule positions are compressed to a single per-molecule centroid by averaging across the exposure window. Molecule states (see below) are compressed by mode (most frequent state during the window); site positions are averaged; site occupancy is the logical OR across the window (a site is marked occupied if it was occupied at any point during exposure). This produces a faithful integration of motion and switching during the camera exposure rather than a single instantaneous snapshot.

#### Molecule states and dynamics

##### State space

The simulator supports two state schemes:

- **3-state**: molecules occupy one of: Free, Intermediate, and Bound states. The Bound state consists of both specific binding to binding sites and non-specific (NS) binding.
- **2-state**: the Intermediate state is removed; only Free and Bound states (both specific and non-specific) exist.

##### Initial populations

The user supplies the initial percentages of mobile molecules in Free, Intermediate, and Non-Specifically bound states. Initial positions are drawn uniformly inside the nucleus and states are sampled from the supplied distribution.

##### Per-state diffusion

At every physics step *Δt*, every mobile molecule is assigned a diffusion coefficient *D* drawn from a Gaussian whose mean and standard deviation depend on its current state. Negative draws are clipped to a small positive value. Specifically bound molecules are pinned to their cognate site for the duration of binding and move along with the site.

##### First-order state transitions

At every *Δt*, mobile molecules undergo Markov transitions at user-specified rates. NS-bound molecules return to the mobile pool with probability *Δt/τ_NS_* where *τ_NS_* is the user-specified mean non-specific residence time (1/*τ_NS_* = *k_off_* or the non-specific off-rate). After unbinding, molecules get assigned to the Free or Intermediate state in proportion to the user-set Free:Intermediate population ratio (in 2-state mode the destination is always Free). Newly transitioned molecules are immediately re-assigned a state-appropriate diffusion coefficient.

##### Specific binding

At each *Δt*, the positions of mobile molecules are queried against a *k-d* tree of binding-site coordinates (rebuilt only when sites have moved). Any mobile molecule within the user-set capture radius of a free site can bind with probability *P_on_*. When cooperative binding is enabled, this probability becomes *p_on_* · [1 + (*αn*)^*h*^] clipped to 1, where *n* is the number of already-bound sites in that cluster, *α* is the cooperativity strength, and *h* is the Hill coefficient. Multiple molecules competing for the same site are resolved by a random shuffle and uniqueness pass such that each site can only be occupied by one molecule. On binding, the molecule is locked to the site position, the cluster's occupancy counter increments, and an unbind time is drawn:

$$t_{res}=t+\max(0.1,N(\tau_{res}\cdot s,\sigma_{res}\cdot S))$$

where *s* is the site strength multiplier and *τ_res_, σ_res_* are user-set residence-time mean and standard deviation. Bound molecules are returned to the mobile pool (Free or Intermediate state) sampled by the same ratio used for NS unbinding.

##### Diffusive step and boundary

Mobile molecules are propagated by an isotropic Gaussian step with per-axis standard deviation $\sqrt{2D\Delta t}$. Any mobile molecule whose updated position lies outside the nuclear sphere is projected radially back onto the surface.

#### Binding-site population

##### Spatial distribution and clustering

A user-specified number of binding sites *N_BS_* are distributed within the nuclear volume following a user-specified cluster-size distribution. For simplicity, the user defines up to five cluster-size bins (binding sites per cluster) and a percentage of total sites in each bin, after which the simulator places clusters whose centres are drawn uniformly at random inside the nucleus. The binding sites for each cluster are drawn uniformly inside a ball of radius *r_cluster_* around the cluster centre. Any sites or centres that would fall outside the nuclear envelope are projected back onto the surface. After the requested clusters are placed, any remaining binding sites are distributed uniformly in the nucleus as singletons.

##### Optional synthetic site array

In addition to the endogenous site population, an optional synthetic cluster (synthetic site array) of *N_syn_* sites can be placed; its centre is drawn uniformly inside the inner 70% of the nuclear radius and its members are drawn uniformly inside a ball of radius *r_syn_* around that centre. The synthetic site clusters are distinctly colored in the ground truth images, and their positions over time are recorded for downstream analysis.

##### Binding-site strengths

Each site is assigned a strength multiplier drawn from a five-bin categorical distribution with user-defined multipliers and bin probabilities. Strengths can be drawn either independently per site or correlated within each cluster (all sites in a cluster receive the same strength). Synthetic sites bypass this distribution and receive the user-set synthetic strength.

##### Site dynamics

Each cluster of binding sites is assigned a diffusion coefficient drawn from a Gaussian with mean and standard deviation specified by the user (clipped at a small positive floor). Cluster centroids then move under fractional Brownian motion (fBm) with a Hurst exponent *H* (the default is *H* = 0.25, showing sub-diffusive Rouse-like polymer motion). *B(t)* is a unique zero-mean Gaussian random variable showing the position of the cluster at time t under fBm. The covariance of the trajectory between time *t* and a future timepoint *s* is given by:

$$Cov[B_{H}(s),B_{H}(t)]=\frac{1}{2}({|s|}^{2H}+{|t|}^{2H}-{|s-t|}^{2H})$$

The discrete increments $\xi_{k}\;=\;B(k\Delta t)-B((k-1)\Delta t)$ form the **fractional Gaussian noise** (fGn), a stationary sequence whose autocovariance at lag *k* (the separation between two increments) is:

$$C(k)=\frac{\sigma^{2}}{2}({|k-1|}^{2H}-2{|k|}^{2H}+{|k+1|}^{2H})$$

where σ^2^ = 2*D*Δ*t* is the per-step variance and *D* is the cluster diffusion coefficient. The normalized autocovariance γ(*k*) = *C*(*k*)/σ^2^ satisfies γ(0) = 1. For *H* = ½, increments are uncorrelated (standard Brownian motion); *H* < ½ yields sub-diffusive (anti-persistent) increments (default); *H* > ½ yields super-diffusive (persistent) increments.

###### Davies–Harte construction.

At each display frame, *n* fGn increments are generated simultaneously for all clusters and all three spatial axes via the circulant embedding of the autocovariance matrix:

1. Form the circulant embedding vector of length *N* = 2*n*:
$$r_{j}=\gamma (j),\;\;0\leq j\leq n;\;r_{j}=\gamma (2n-j),\;\;n<j<2n$$
2. Compute eigenvalues *λ*_j_ = Re[DFT(*r*)]_j_; clip any negative values to zero.
3. Construct random vector *W* of length *N* from independent standard normals *Z*:
$$\;W_{0}=\sqrt{\frac{\lambda_{0}}{N}}Z_{0},\;W_{n}=\sqrt{\frac{\lambda_{n}}{N}}Z_{n}\;W_{j}=\sqrt{\frac{\lambda_{j}}{2N}}(Z^{\left(1\right)}+iZ^{\left(2\right)}),\;\;j=1,\ldots ,n-1;\;W_{N-j}={\underline{W}}_{j}$$
4. The first *n* elements of Re[DFT(*W*)] are exact unit-variance fGn samples {*y*_k_}.
5. Scale to physical units: Δ*x*_k_ = σ *y*_k_ = √(2*D*Δ*t*) *y*_k_.

###### Cross-frame conditioning.

A Davies-Harte block drawn independently at each frame boundary would break the global autocorrelation structure of fBm. The first increment of each new frame block is therefore conditioned on the terminal increment of the previous frame Δ*x*_−1_ via the exact conditional distribution of a stationary Gaussian process (Gaussian bridge):

$$\Delta x_{0}^{bridge}=\gamma_{1}\;\Delta x_{-1}+\sqrt{1-{\gamma (1)}^{2}}\;\sigma ,Z,\;Z∼N(0,I_{3})$$

where γ(1) = ½(2^2H^ − 2) is the lag-1 normalized autocovariance. The remaining Davies-Harte increments are mean-corrected by the kriging update:

$$\Delta x_{k}^{cond}=\Delta x_{k}^{DH}+\gamma (k)(\Delta x_{0}^{bridge}-\Delta x_{0}^{DH}),\;k=1,\ldots ,n_{f}$$

This approximation preserves the long-range autocorrelation structure of fBm across frame boundaries while keeping memory bounded to a single step.

On top of cluster motion, every individual site receives an independent Gaussian “jiggle” at every physics step, to reflect changing conformations of binding sites that are close to each other. Sites are constrained to remain at least one capture radius inside the nuclear surface so that no capture sphere is clipped by the boundary.

#### Image formation

##### Voxelization and point-spread function

For each output frame the recorded positions across the exposure window are concatenated and binned onto an isotropic 3D grid with a user-set voxel size, corresponding to the detector pixel size. Each binned position is convolved with an anisotropic 3D Gaussian point-spread function (PSF) with separately specified lateral and axial full-widths at half maximum (*FWHM_xy_, FWHM_z_*). Standard deviations are obtained as $\sigma =FWHM∕(2\sqrt{2ln2})$. The PSF kernel is truncated at *3σ* in each axis and normalized to unit mass before deposition such that each molecule represents 1 intensity unit. Voxels falling outside the nuclear sphere are set to zero.

##### Z-slice projection

The 3D voxel grid is sliced into 2D z-planes spaced at the user-set inter-slice distance. Each slice is the single voxel layer at the z-coordinate (no axial integration beyond what the PSF already provides). Slices are saved as multi-page TIFFs.

##### Intensity normalization

Two mutually exclusive normalization modes are supported:

1. **Auto-normalize.** All rows are simulated and a single global $v_{max}$ equal to max voxel intensity across every frame is then used to normalize every output. This guarantees intensity comparability across rows. A noise floor as a percentage of the $v_{max}$ can be added. For the batch mode, the normalization is across all conditions batched. A headroom factor can be added for visualization.
2. **Arbitrary-units (AU) calibration.** When AU mode is enabled, the 3D grid is rescaled so that the mean intensity inside the nuclear mask matches an expected signal $S=(c∕c_{ref})$ derived from a user-supplied reference concentration-signal pair. A constant background and Gaussian noise are added inside the nucleus. Output is clipped to the chosen bit depth. AU calibration is set per row; rows with it enabled override the global brightness mode.

##### Output products

Three image products may be saved per frame (as TIFFs):

- **Convolved.** The PSF-convolved z-slice stack described above, downloaded as 8- or 16- bit. This is the primary simulated microscopy output.
- **Ground truth.** A scatter plot of true molecule centroids (coloured by state) overlaid with binding-site positions (coloured by strength, with synthetic sites highlighted), rendered at a user-specified upscale factor for visual inspection.
- **Molecule count map.** A z-slice stack containing, per voxel, the raw count of molecules present there during the exposure window — the same geometry as the convolved stack but without PSF convolution. Three count modes are provided: *full exposure* (every sub-step during the exposure window contributes one count per molecule, so a molecule present for *N* sub-steps is counted *N* times), *snapshot* (only the final sub-step is counted, giving instantaneous voxel occupancy) and centroid (an average of the sub-steps is shown). Two z-plane projection modes are provided: *z-slab* (voxels within ±(slice-spacing)/2 of each z-slice are summed) and *single voxel* (only the single layer at the z-slice is reported).

A CSV containing the positions and state of each molecule in each frame is also available to download. When a synthetic site array is present, a centroid CSV is also written, giving the centre-of-mass of the synthetic cluster. Finally, a .pkl file that saves the rendered images can be downloaded, allowing users to upload it to perform FRAP at a later time.

#### Reproducibility

To support exact replay and to support comparing nominally identical conditions across rendering parameters, the simulator has an integer-seed mechanism that makes every random draw — site positions, strengths, per-cluster diffusion coefficients, fBm increments and jiggle, initial molecule placements, state transitions, binding dice, and residence-time draws — deterministic.

##### Interactive mode

A "Use random seed (reproducible)" checkbox in the General Parameters panel exposes an integer seed input. When checked, the engine calls numpy.random.seed(seed) once at entry, before any random draw. The user can choose whether to freeze the trajectories (visualization parameters can be changed) or only the binding site characteristics (molecule kinetic parameters can also be changed).

##### Batch mode

Per-row reproducibility is controlled by three optional CSV columns:

- **sim_seed —** integer random number generator seed for the row.
- **sim_group —** string label tying together rows that should share *everything* (sites, motion, binding events). Only rendering parameters (PSF FWHMs, voxel size, slice spacing, bit depth, brightness, AU parameters) may differ within a sim_group.
- **site_group —** string label tying together rows that should share *only* the site layout (positions, cluster IDs, strengths, per-site diffusion coefficients). Motion physics — concentration, kinetics, diffusion coefficients — may vary within a site_group. If site_group is blank, it is auto-derived from sim_group.

The per-row seed is resolved as: (1) the row's sim_seed cell, if a usable integer; otherwise (2) base_seed + row_index, where base_seed is the UI-supplied base integer (mirroring the SLURM --seed convention); otherwise (3) operating-system entropy. Because every random draw inside the engine is reached deterministically from a single global seed, two rows that share a sim_group and seed produce identical output, and two rows that share a site_group and seed produce identical sites with independent motion — the same operational guarantee as the HPC implementation, achieved without an explicit cache. The seed used for every row is logged to seeds_used.csv inside the output ZIP.

#### FRAP Module

This allows the user to FRAP a cluster in a simulated nucleus. The molecules in the FRAP user-defined spherical region-of-interest are turned to an irreversible dark, “bleached” state. A recovery curve is generated, measuring the intensity within the same region. The post-FRAP images and intensity values are available to download.

#### Batch processing

A batch run is driven by a CSV in which each row is a complete parameter set. A pre-filled template is generated by the application from the same defaults used by the interactive mode, with the optional reproducibility columns (sim_seed, sim_group, site_group) appended at the end. Rows are processed sequentially and outputs are bundled into a single downloadable ZIP with a top-level subfolder per row. When auto-normalize brightness mode is selected, the simulator runs in two phases: all rows are simulated and gridded in phase 1 to establish the global maximum intensity; phase 2 then renders all rows against that single $v_{max}$ so that intensities are directly comparable across the entire batch.

#### HPC implementation

The same physics is also implemented as a standalone command-line tool for parallel processing on an HPC. There are two operational differences:

1. The HPC tool uses one SLURM array task per CSV row, so all rows run in parallel under independent processes. The application processes rows sequentially in the browser session. Because every row is fully determined by its own seed and parameters, the two execution orders produce the same per-row output.
2. The HPC tool implements an explicit ground-truth cache: the first row in each sim_group writes its full simulation to disk and the subsequent rows in the same group skip simulation entirely and re-render from the cache. The application does not implement this cache: every row is re-simulated. However, because seeded rows are deterministic, the *output* of sim_group and site_group membership is preserved — rows that share a sim_group and seed produce identical output in the application; rows that share a site_group and seed produce identical sites with independent motion. The application is thus slower than the HPC tool on grouped batches but produces matching results.

### Simulations generated for this manuscript

All simulations, except for the FRAP and fusion examples, were generated using the HPC version of SPARK. Dorsal concentrations on the ventral side of the embryo (500 nM) were obtained from published work^13^ and the concentrations on the medial (406 nM) and lateral (219 nM) sides were estimated through intensity calibrations. The molecules were modelled with cooperative binding with a Hill coefficient of 100, as detailed previously^10^. Bicoid concentrations along the anterior-posterior gradient (20%: 25 nM, 30%: 16 nM, 40%: 11 nM, 50%: 7 nM, and 60%: 3.5 nM) were estimated from published work^17^. Bicoid clusters in SPARK-generated images were segmented using a previously-published custom segmentation pipeline developed in the lab^9,10^ and were overlaid on the molecular count images to obtain the number of molecules per cluster. Five nuclei were simulated for each concentration and the results were pooled. Sox2 concentrations and the clustering of binding sites in mouse ES cells were obtained from published work^20,21^. Renderings were generated from the positions of only the specifically bound molecules. Virtual FRAP was done for a simulated protein with BRD4-like characteristics (high concentration, large number of binding sites), and intensities were normalized to the pre-bleach intensity within the bleached ROI. Many examples of cluster fusion were seen across various simulations - the displayed example is for the BRD4-like protein.

Full parameter sets for each displayed example can be found and reused from the dropdown menu of the GUI, or in the “interactive_presets.csv” present in the zipped folder.

### Imaging

#### Fly lines used

The fly line used for bicoid imaging was *yw*; *his2av-mrfp1; BcdE1, egfp-bcd*. Dorsal images are from heterozygous progeny from the lines *yw; Dorsal-mNeonGreen/Cyo;* and *nos>MCP-mCherry (III)* crossed with males containing an MS2 reporter.

#### Embryo preparation for live imaging

Drosophila embryos were manually dechorionated by gently rolling them on double-sided tape using a blunt needle. Dechorionated embryos were arranged on an agar pad and transferred onto an adhesive heptane-acrylic spot on a 25mm glass coverslip (lattice light-sheet) or the hydrophobic side of a semi-permeable membrane in a 3D printed sample holder (benchtop confocal).

#### Lattice light-sheet imaging

Lattice light-sheet volumetric imaging of Bicoid was performed on a Multimodal Optical Scope with Adaptive Imaging Correction (MOSAIC)^22^. The 488 nm excitation laser was used at 10% (0.552 mW at the back focal plane of the excitation objective) to excite eGFP. A volume of 18.9 μm sampling z-planes spaced 0.3 μm apart was acquired at various positions along the anterior-posterior axis of the embryo, with 30 ms exposure time. Dorsal images were obtained from previously published work^10^.

#### Spinning disk confocal imaging

Confocal imaging was performed on an Andor BC43 Spinning Disk Confocal Microscope with a 20x/0.8 NA objective to obtain a full-embryo image. The 488 nm excitation laser was used at 15% (16.9 mW) to excite eGFP. A volume of 34 μm sampling z-planes spaced 0.3 μm apart was acquired, with 100 ms exposure time.

### Image Analysis

Lattice light-sheet images were pre-processed using GPU-accelerated 3D deconvolution via CUDA^23^. Deconvolution was done using a Richardson-Lucy based algorithm with 5 iterations, utilizing point spread functions (PSFs) captured from bead images acquired on the day of imaging to ensure accurate correction.

All images were processed, scaled and visualized in FIJI or Napari. Displayed kymographs were constructed by taking a 600 nm maximum projection across z-slices, generating a kymograph for each column in the red rectangle, and max-projecting the 5 resulting kymographs.

**Fig. 2:**
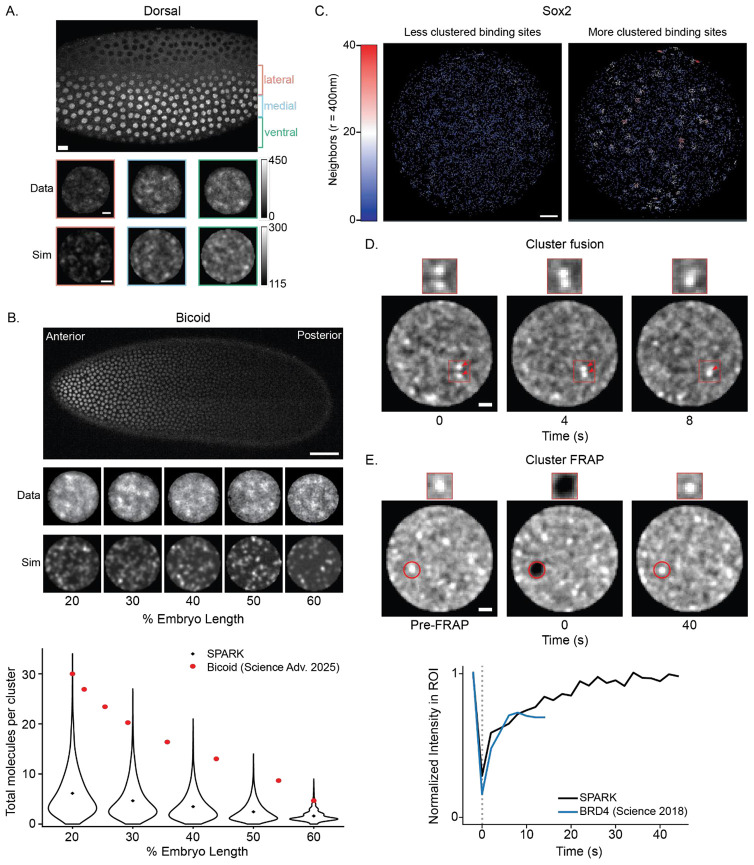
Comparison of simulations to experimental data. **(A)** Dorsal gradient. Top: Maximum intensity projection image showing nuclear gradient of Dorsal-mNeonGreen in a Drosophila embryo in nc13. Scale bar is 15 μm. Bottom: Data vs. simulated images of individual nuclei (single frame, single z-slice). **(B)** Bicoid gradient. Top: Maximum intensity projection image of Bicoid-GFP in a Drosophila embryo in nc14. Scale bar is 50 μm. Middle: Data vs. simulated images of individual nuclei (single frame, single z-slice). Images are contrast-adjusted to highlight clusters. Bottom: Number of Bicoid molecules per hub along the Bicoid gradient. **(C)** Density rendering of bound Sox2 binding in ES cells. Scale bar is 1 μm. **(D)** Sample images showing clusters exhibiting fusion. Insets show zoomed-in views. Scale bar is 1 μm. **(E)** Sample images (left) and intensity trace (right) for a simulated FRAP experiment. Red circle depicts a 1 μm diameter photobleached region in which intensity is measured. Insets show zoomed-in views of the red circles. Scale bar is 1 μm.

Data from other papers (Fig. 2) was directly approximated from figures using PlotDigitizer^24^.

### Software Development and AI Assistance

SPARK was implemented in Python by the authors with the assistance of a large language model (Anthropic Claude Opus, online and through Claude Code). Claude was used for code generation, GUI creation, editing, and for the suggestion and implementation of algorithms such as the Davies–Harte construction and cross-frame conditioning for approximate fractional Brownian motion. All AI-assisted code and text were reviewed, tested, and validated by the authors, who take full responsibility for the correctness of the implementation.

## Supplementary Material

Supplement 1

Supplement 2

Supplement 3

Supplement 4

## Figures and Tables

**Fig. 1:**
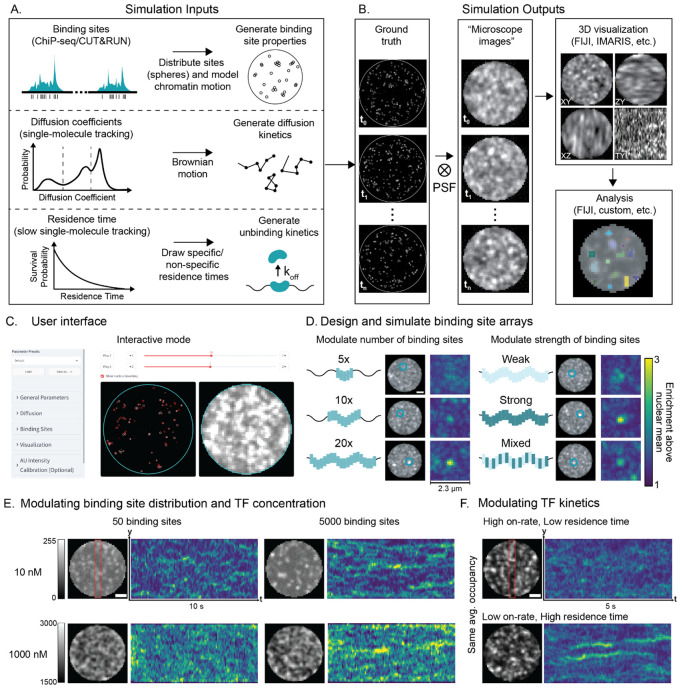
Overview of simulation software. **(A)** Simulation inputs. Users input binding site characteristics, molecular diffusion coefficients, and residence times. **(B)** Simulation outputs. The simulator generates molecular positions which are convolved with a user-defined point spread function (PSF) to obtain z-slices of “microscope images” at each time point. These can be visualized in any image analysis software and further analysed. **(C)** Screenshot of user interface. **(D)** Simulating binding sites with varying numbers/strengths. Left: Binding sites schematic. Middle: Simulated nucleus. Blue circle indicates binding site array position. Right: Enrichment of TF over nuclear background, centered at the binding array. **(E)** Nucleus image (left) and kymographs over time (right) showing the effects of changing nuclear concentration and the number of binding sites. Scale bars are 1 μm. **(F)** Nucleus image (left) and kymographs (right) in the max-projected red area showing the effects of changing the TF on-rate and residence times. Scale bars are 1 μm.

## Data Availability

All simulated data can be reproduced using the provided code.

## Code Availability:

Developed code is available on Zenodo: https://doi.org/10.5281/zenodo.20533508.
