# Supplementary material for: Simulation of transcription factor clustering in nuclei from molecular kinetics
Source: bioRxiv. 2026 Jun 6:2026.06.04.730171. Preprint. [Version 1] doi: 10.64898/2026.06.04.730171 (PMC13252165; doi:10.64898/2026.06.04.730171)
Supplement: Supplement 1 [file media-1.pdf]

A.

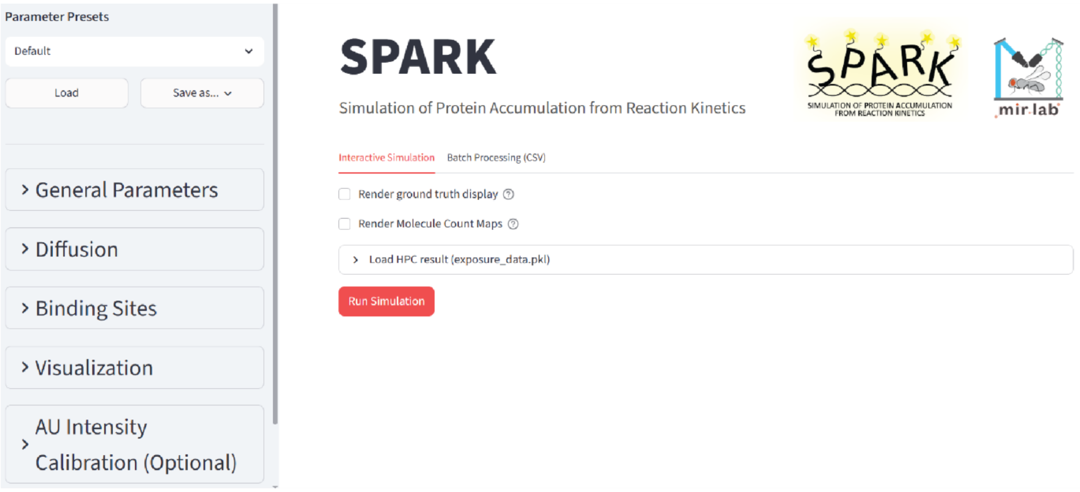

B.

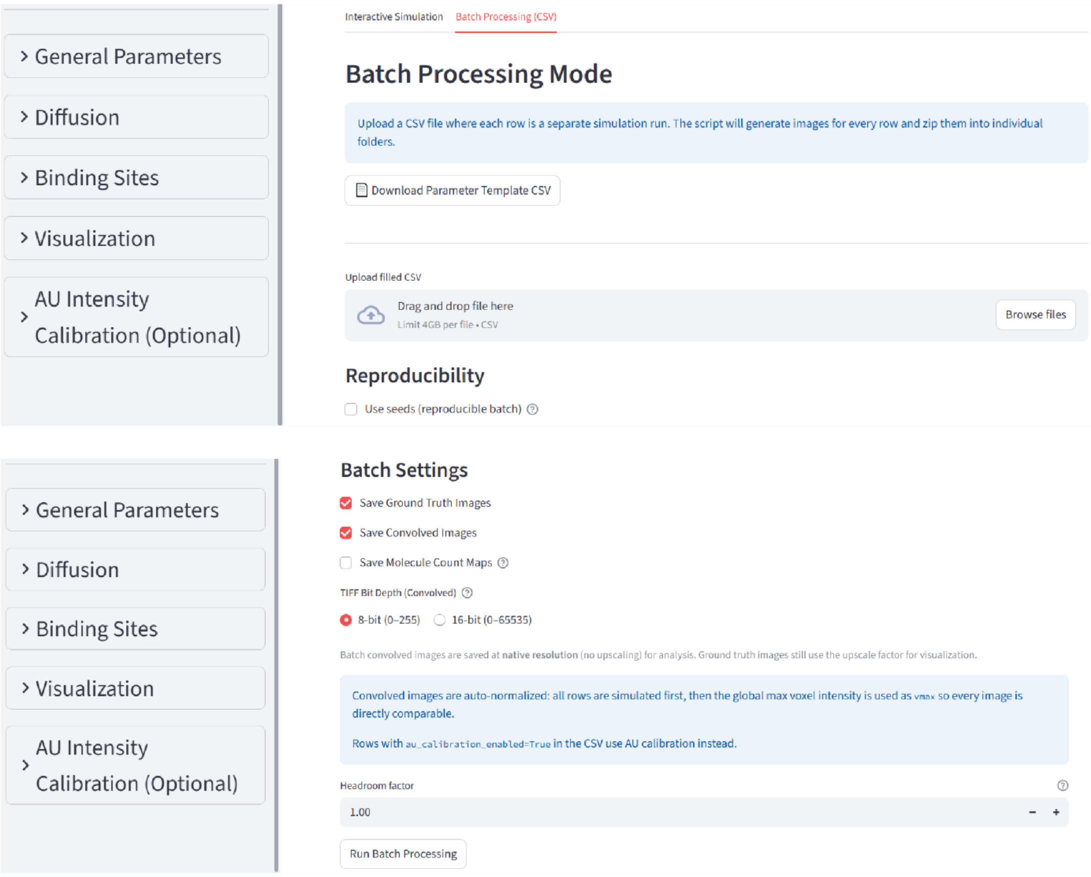

Fig. S1: Screenshots of GUI in either (A) Interactive or (B) Batch Processing mode.

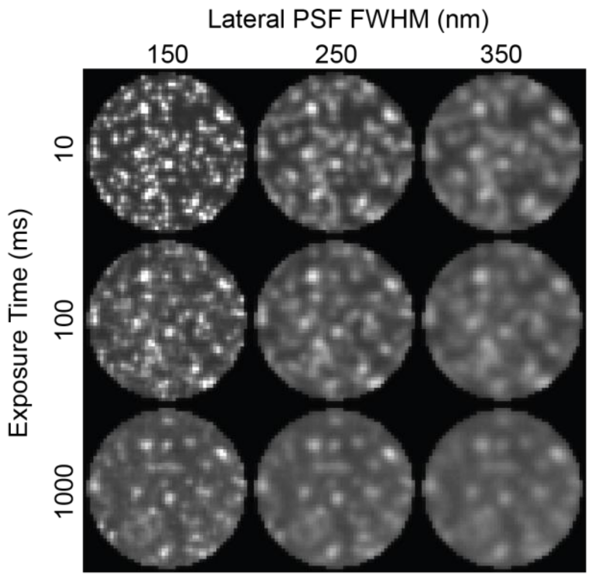

Fig. S2: Single timepoint, single optical slice images of nuclei with identical molecular kinetics, showing the effect of modulating either the lateral point spread function or the exposure time.

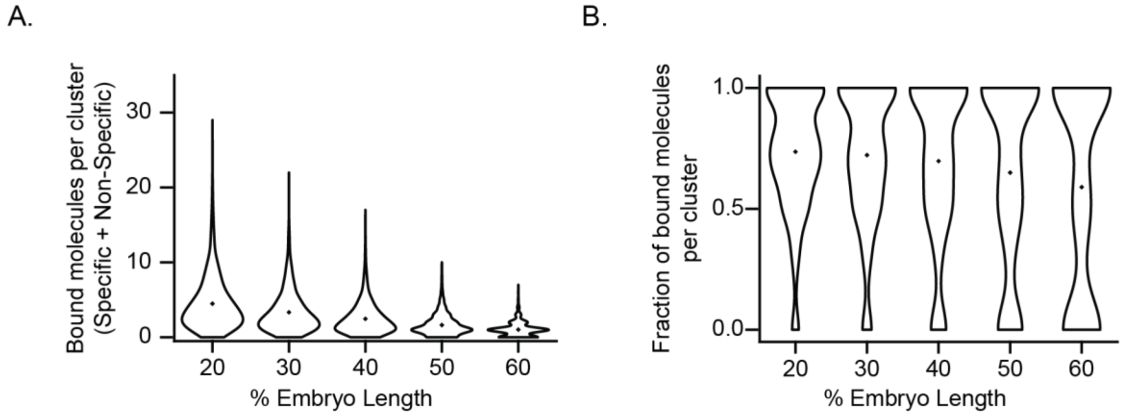

Fig. S3: (A) Number and (B) fraction of bound bicoid molecules per cluster at various positions along the embryo length. Bound molecules include both specific and non-specifically bound molecules.
