# Supplementary material for: Simulation of transcription factor clustering in nuclei from molecular kinetics
Source: bioRxiv. 2026 Jun 6:2026.06.04.730171. Preprint. [Version 1] doi: 10.64898/2026.06.04.730171 (PMC13252165; doi:10.64898/2026.06.04.730171)
Supplement: Supplement 2 [file media-2.pdf]

Supplementary Movie Captions

Supplementary Movie 1: 3D rendering of specifically-bound Sox2 molecules in mouse ES cell nuclei with non-clustered (left) and clustered (right) binding sites.

Supplementary Movie 2: Simulated FRAP analysis from Fig. 2E. The red circle indicates the bleached area. The duration of the movie is 60 sec. The movie was rendered at 5 fps.
